# Detection and genetic characterization of Tembusu virus and other flaviviruses from mosquitoes in Lao PDR

**DOI:** 10.1371/journal.pone.0351023

**Published:** 2026-06-08

**Authors:** Chittaphone Vanhnollat, Somsanith Chonephetsarath, Somphavanh Somlor, Veaky Vungkyly, Tiger Soulaphy, Sopha Vongsanga, Irina V. Etobayeva, Thomas Bigot, Gary Wong, Andrew G. Letizia, Paul T. Brey, Philippe Buchy, Khamsing Vongphayloth

**Affiliations:** 1 Institut Pasteur du Laos, Laboratory of Virology, Vientiane, Lao PDR; 2 Institut Pasteur du Laos, Laboratory of Vector-Borne Diseases, Vientiane, Lao PDR; 3 Lao Army Institute for Disease Prevention, Vientiane, Lao PDR; 4 Science Directorate, U.S. Naval Medical Research Unit INDO PACIFIC, Sembawang, Singapore; 5 Bioinformatics and Biostatistics Hub, Institut Pasteur, Université Paris Cité, Paris, France; 6 Institut Pasteur du Laos, General Director, Vientiane, Lao PDR; CEA, FRANCE

## Abstract

**Background:**

Lao People’s Democratic Republic (Lao PDR), located in Southeast Asia and known for its rich biodiversity, is part of a region recognized as a hotspot for emerging and re-emerging infectious diseases. Among flaviviruses, dengue virus (DENV) and Japanese encephalitis virus (JEV) are recognized public health threats. However, other reemerging mosquito-borne flaviviruses may also infect humans and cause diseases. Despite that, their distribution and public health impact in Lao PDR are not well understood due to limited past surveillance.

**Methodology:**

Mosquitoes were collected using CDC light traps from 2021 to 2024, as part of vector and pathogen surveillance studies conducted across six provinces. A total of 2,548 female mosquitoes, representing 100 species from 11 genera, were collected and morphologically identified. Of these, 1,622 mosquitoes were pooled into 1,008 “mini pools” according to species and collection site. The pools were screened for flaviviruses by nested RT-PCR. Positive samples were further analysed by metagenomic sequencing, and coding-complete genomes were recovered and subjected to phylogenetic analysis.

**Primary results:**

We recovered thirteen coding-complete genomes through metagenomic sequencing, which included one Tembusu virus (TMUV) strain (TMUV/Mos_L010) from *Culex vishnui* mosquitoes and 12 other insect-specific flaviviruses (ISFVs). Phylogenetic analysis placed TMUV/Mos_L010 in cluster 3, closely related to a TMUV strain known to be pathogenic to dolphins in Thailand, with more than >99% bootstrap support for amino acid homogeneity. The detected ISFVs were part of the classical insect-specific flavivirus (cISFV) lineage and were further classified into five subgroups according to their associated mosquito genera: *Aedes* (1), *Anopheles* (1), *Culex* (2), and *Uranotaenia* (1).

**Conclusions:**

This study documents the first detection of TMUV in Laotian mosquitoes and extends the known distribution of cluster 3 TMUV strains. The discovery of diverse ISFVs shows the rich and underexplored virome among Laotian mosquito populations. These findings highlight the need for enhanced arbovirus surveillance and ecological research to assess zoonotic risks of spillover infections in Southeast Asia.

## Introduction

Flaviviruses, belonging to the family *Flaviviridae* and genus *Orthoflavivirus*, are a diverse group of enveloped, positive-sense, single-stranded RNA viruses with genomes approximately 9.0–13 kb in size, many of which are host-specific [1]. Most flaviviruses are arthropod borne, with several posing significant threats to human and animal health. Dengue virus (DENV), Yellow fever virus (YFV), Zika virus (ZIKV), West Nile virus (WNV), and Japanese encephalitis virus (JEV) are well-studied flaviviruses that cause severe morbidity and mortality in humans and animals [2].

Flaviviruses are primarily transmitted to humans by *Aedes* and *Culex* species of mosquitoes. *Aedes*-associated flaviviruses, YFV, DENV, and ZIKV, and *Culex*-associated flaviviruses, WNV and JEV, have long been responsible for recurrent human epidemics. Notably, DENV, YFV, WNV, and ZIKV collectively cause approximately 400 million cases globally each year [3,4]. *Aedes*-associated flaviviruses present a diverse range of symptoms depending on the specific pathogen. DENV can result in severe manifestations such as haemorrhagic fever and shock syndrome. YFV is characterized by jaundice and can progress to liver failure. ZIKV is associated with congenital anomalies like microcephaly and neurological conditions like Guillain-Barre Syndrome. In contrast, *Culex*-associated viruses typically exhibit neurotropic effects, leading to severe neurological sequelae and potential death. Uniquely, ZIKV, despite being *Aedes*-associated, is both neurotropic and the only known flavivirus capable of sexual transmission [5].

Beyond these well-characterized pathogens, emerging flaviviruses pose increasing risks to human and animal health [6]. Several mosquito-borne flaviviruses, including Usutu virus (USUV), Wesselsbron virus (WSLV), Spondweni virus (SPOV), Ilheus virus (ILHV), Rocio virus (ROCV), and Murray Valley encephalitis virus (MVEV), have varying degrees of zoonotic potential and neurotropism. For instance, USUV, is expanding in Europe, affecting birds and occasionally causing neurological diseases in humans [7]. MVEV, which is endemic to Australia and Papua New Guinea, has been responsible for sporadic encephalitis outbreaks [8]. The processes of globalization and urbanization are facilitating the spread of these flaviviruses and their vectors, thereby increasing the risk of disease emergence in new regions [4].

Tembusu virus (TMUV) is an emerging flavivirus that has significantly affected the poultry industry in China and Southeast Asia [9]. First isolated in 1955 from mosquitoes in the Malaysian Peninsula [10], TMUV causes severe egg drop syndrome in laying ducks and neurological disorders with high mortality rates in ducklings [11]. The virus has been identified in various avian species, including chickens, geese, pigeons, and sparrows, causing substantial economic losses [12].

While TMUV primarily affects avian species, recent studies have shown its ability to infect mammals, including mice [13] and dolphins [14], causing neurological pathology and death. Likewise, various mammalian and human-derived cell lines have been found susceptible to TMUV infection [15]. TMUV RNA and antibodies have been detected in humans, raising concerns about its zoonotic potential [16,17], although infectious virus has not yet been isolated from human cases.

The Lao People’s Democratic Republic (Lao PDR), located on the Indochinese Peninsula, is a biodiverse country with different ecosystems [18]. Although the country lies within a region recognized as a hotspot for emerging and re-emerging infectious diseases [19], its limited and under-resourced health care system [20] hinders effective prevention and control of emerging infectious diseases. There is a notable lack of arbovirus surveillance data, particularly concerning mosquito vectors and circulating emerging flaviviruses. To address this gap and enhance local scientific capacity, the Institut Pasteur du Laos (IPL) has collaborated with the U.S. Naval Medical Research Unit INDO PACIFIC (NAMRU INDO PACIFIC) to establish a risk-based surveillance network aimed at the early detection of vector-borne pathogens. This initiative has facilitated collection and analysis of data on arbovirus detection in field-caught mosquitoes from various regions of Lao PDR. The primary objective of this study was to implement a risk-based arboviral surveillance system in Lao PDR and to assess the diversity of flaviviruses in mosquito populations across different ecological habitats. This work resulted in the detection and genetic characterization of Tembusu virus (TMUV) in Culex mosquitoes, which was an unexpected finding during the surveillance activities.

## Materials and methods

### Mosquito collection and identification

**Fig 1:**
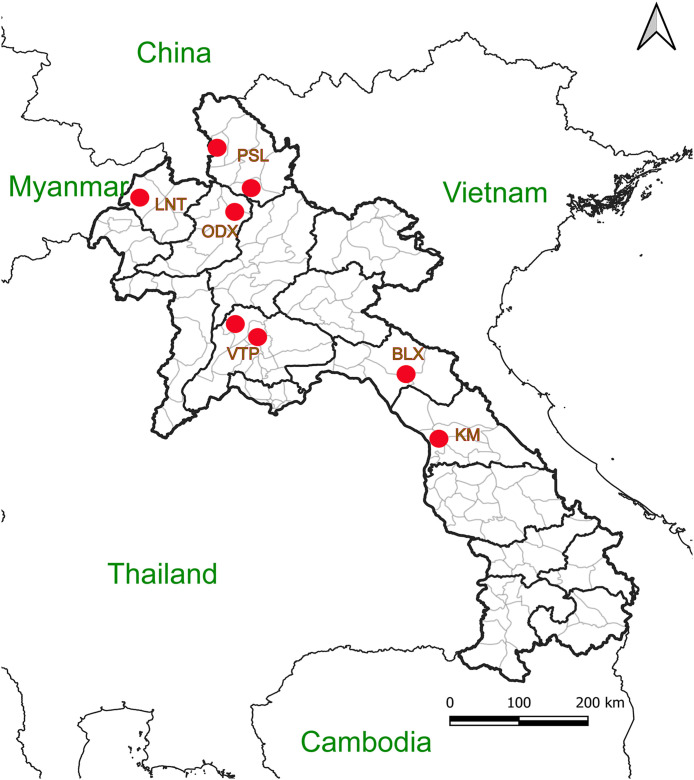
The map shows the locations (red dots) where mosquitoes were collected for this study. The regions are designated as follows: BX – Bolikhamxay, KM – Khammouane, LNT – Luangnamtha, ODX – Oudomxay, PSL – Phongsaly,and VTP – Vientiane province. The map was created using QGIS software with open-source and public domain data (Natural Earth).

Standard collection methods using the Center for Disease Control and Prevention (CDC) light traps were used to collect mosquitoes from different types of habitats described below. The traps were set between 4:00–6:00 p.m. and 8:00–9:00 a.m. of the following day. Specimens were collected between 2021 and 2024 and stored at −80⁰C in the IPL repository. Mosquitoes were collected from six provinces: Bolikhamxay, Khammouane, Luangnamtha, Oudomxay, Phongsaly and Vientiane province (Fig 1).

Collection periods varied by site and year. Field missions were conducted at the following time points: July–August 2021 in Xay District (Oudomxay); December 2022 in Bounneua and November 2023 in Khua (Phongsaly); January, June, and July 2023 in Viengthong (Bolikhamxay); August and December 2023 in Thakhaek (Khammouane); March, June, August, and October 2023 in Kasi and September and December 2023 in Vangvieng (Vientiane Province); and May 2024 in Long (Luangnamtha).

Sampling sites were selected to represent a range of ecological settings, including karstic cave areas, forests, agricultural plantations (e.g., rubber and orange), and domestic animal shelters in rural villages. The frequency of mosquito collection was not uniform across sites, as it depended on field accessibility, seasonal conditions, and specific project objectives. Locations varied between years to expand geographic coverage under the same surveillance program. Geographic coordinates were recorded for each sampling site and are summarized in S1 Table.

The sampling sites in this study were classified into the following categories: (i) karstic cave/karstic areas, (ii) rubber plantation, (iii) orange plantation, (iv) forest path areas, and (v) domestic animal sheds in villages (S1 Table). Most collection sites, apart from those in Oudomxay and Phongsaly provinces, were in karstic mountain regions, where villagers frequently visited caves for various activities.

The sampling site map was created using QGIS software and open-source data. The Lao administrative shapefile data was downloaded from Natural Earth (public domain: https://www.naturalearthdata.com/).

The mosquito samples collected with CDC light traps were placed in −20°C for at least 30 minutes, followed by sorting and counting on ice packs to maintain a stable low temperature. Specimens were identified to the genus and, when possible, to species level under a stereo microscope using morphological taxonomic keys [21–25]. All samples were transported from the field to IPL on dry ice or liquid nitrogen for arbovirus isolation attempts.

### Sample preparation, RNA extraction and virus screening

Between 1–10 mosquitoes were pooled according to genus, species and geolocation to form a “mini pool”, then homogenized for 5−10 min in 0.7 mL of 1x phosphate-buffered saline (PBS) with the Lysing Matrix E zirconium beads (MP Biomedicals) using a TissueLyser II system (Qiagen). Total nucleic acids (DNA and RNA) were extracted from 150 µl of clarified supernatant using the NucleoSpin8 extraction kit (Macherey-Nagel) following the manufacturer’s protocol. Purified DNA and RNA were pooled to form a “big pool” of eight “mini pools” and screened for arboviruses using nested RT-PCR with generic targets per methods described by Sanchez-Seco *et al.* (2005) for identification of pathogenic flavivirus [26]. The first round used primer Flavi1_F (5’-GAYYTIGGITGYGGIIGIGGIRGITGG-3’) and Flavi1_R (5’-TCCCAICCIGCIRTRTCRTCIGC-3’), while the nested round used Flavi2_F (5’-YGYRTIYAYAWCAYSATGGG-3’) and Flavi2_R (5’-CCARTGITCYKYRTTIAIRAAICC-3’). DENV was used as a positive control. When a “big pool” tested positive, each “mini pool” was further re-tested by nested RT-PCR. Positive PCR products were sequenced using Sanger sequencing. The maximum likelihood (ML) estimates of the prevalence were calculated using results of the “big pool” that was tested using PoolTestR package for the R language [27].

### Virus isolation

One Tembusu virus-positive “mini pool” of 10 mosquitoes homogenized as described above was used to attempt virus isolation. The virus isolation attempt was performed using BHK21 and Vero E6 cells. The cells were cultured with Dulbecco’s Modified Eagle Medium (DMEM, Gibco) supplemented with 5% Fetal Bovine Serum (FBS, Gibco) and 1% antibiotic solution containing Streptomycin and Penicillin (Gibco) until the cells reached 70–80% confluency. A volume of 500μl of sample supernatant was filtered using a 0.22 μm filter (Sartorius). The filtered sample was added to 25 T culture flasks containing a confluent monolayer of cells and pre-filled with 1 ml of DMEM. The inoculated flasks were incubated at 37°C with 5% CO_2_ for 1 h. Following the incubation, 4 ml DMEM supplemented with 5% FBS and 1% antibiotic were added and further incubated at 37 °C with 5% CO_2_. The inoculated flasks were observed and recorded daily for 7 days, followed by 3 additional blind passages. Culture supernatants and cell layers were collected on days 3, 5, and 7 and tested for flavivirus detection using RT-PCR, as described above.

### NGS library preparation and sequencing

Overall, 19 positive “mini pools” underwent Next Generation Sequencing (NGS). Genomic libraries were prepared using the SMARTer Stranded Total RNA-seq kit v3-Pico input mammalian kit (Takara Bio) according to the manufacturer’s recommendations. The Qubit DNA High sensitivity assay (Invitrogen) and the Agilent High Sensitivity D1000 ScreenTape System (Agilent Technologies, Inc.) were used, respectively, for quantification and quality controls of the libraries. Prepped libraries were then sent to the Macrogen APAC, Singapore, for sequencing on the NovaSeq X Plus in a 2 × 150 bp paired-end format to achieve a minimum of 2 GB of data for each library.

### Sequencing data analysis

The Microseek bioinformatics pipeline was used for virus assignment [28]. Briefly, after quality check and trimming of raw reads, *de novo* assembly of reads was performed using Megahit [29]. Resulting contigs and singletons were translated into protein sequences across all six reading frames using an in-house program. Sequences shorter than 15 amino acids were removed, and taxonomic assignment of all sequences was performed using the DIAMOND [30] and BLAST tools with three successive databases: i) reference viral protein database, RVDB-prot [31], ii) generalist protein database, NCBI/nr, and iii) generalist nucleotide database, NCBI/nt. The open reading frames (ORFs) of each sequence were identified using a standalone version of the NCBI’s ORFfinder (https://www.ncbi.nlm.nih.gov/orffinder/, accessed on 22 March 2025). The protein domain identification of the resulting ORFs was then performed using the InterProScan 5.73–104.0 [32]. The mean coverage of contigs was performed using the Koverage with the mapping-based method [33].

### Phylogenetic and sequence similarity analysis

Sequence alignment and removal of ambiguously aligned regions were performed using the MAFFT [34] and BMGE [35], respectively. The ML trees were constructed using the IQ-TREE version 2.3.6 [36] with 1000 ultrafast bootstrap replicates. The best-fit model of substitution was identified using the ModelFinder [37]. Finally, the phylogenetic trees were visualized and annotated using the Tree Visualization By One Table (tvBOT) [38]. The identity matrices of nucleotide (NT) and amino-acid (AA) sequences were calculated using FastANI [39] and EzAAI [40], respectively. Sequence similarity analyses were performed using the SimPlot++ [41].

## Results

### Mosquito diversity and species composition

A total of 2,548 female mosquitoes were collected and morphologically identified into 109 species across 11 genera, including *Aedes* (14), *Anopheles* (30 species), *Armigeres* (12), *Culex* (22), *Lutzia* (1), *Malaya* (1), *Mansonia* (5), *Mimomyia* (1), *Topomyia* (1), *Tripteroides* (1), and *Uranotaenia* (21) (S2 Table). When compared to previous literature regarding mosquito species in Lao PDR, 35 additional species were identified, updating local records and checklists to include a total of 205 species [42].

Most mosquitoes in this study were collected from village sites, which also represented the majority of sampling locations (n = 1,630; species richness r = 74), followed by cave/karstic areas (n = 638; r = 67), forests (n = 131; r = 36), orange plantations (n = 113; r = 18), and rubber plantations (n = 36; r = 16). The most frequently encountered species in village areas were the *Culex vishnui* group (n = 808), followed by the *Anopheles minimus* (n = 152) (S2 Table). Analysis of monthly species composition across different districts and provinces showed substantial seasonal variation in mosquito diversity. Both mosquito abundance and species diversity fluctuated throughout the year, with peak diversity observed in the wet season (May-October), while in the dry season (November-April) only few species persisted year-round (S1–S3 Figs).

### Initial virus screening

A subset of 1,622 out of 2,548 mosquitos were pooled into 1,008 “mini pools” and screened. A total of 19 out of 1,008 (1.88%) tested positive for flaviviruses (see Table 1).

**Table 1:** Mosquitoes positive for flaviviruses by pan-flavivirus RT-PCR.

Mosquito Genera	Total N° of Pools	Total N° of Mosquitoes	N° of Positive Pools (%)	N° of Mosquitoes in Positive Pools	MLE^#^ (95% CI)
*Aedes*	74	86	3 (4.05)	3	3.49 (0.879-8.80)
*Anopheles*	67	121	4 (5.97)	14	3.45 (1.084-7.85)
*Armigeres*	79	153	1 (1.27)	4	0.66 (0.038-2.87)
*Culex*	486	892	7 (1.44)	17	0.79 (0.340-1.52)
*Lutzia*	1	1	0	0	0.00 (0.000-85.35)
*Malaya*	2	2	0	0	0.00 (0.000-61.72)
*Mansonia*	58	63	0	0	0.00 (0.000-3.00)
*Mimomyia*	3	3	0	0	0.00 (0.000-47.28)
*Topomyia*	1	1	0	0	0.00 (0.000-85.35)
*Uranotaenia*	169	224	4 (2.37)	7	1.80 (0.562-4.13)
Whole_body_not_ID*	68	76	0	0	0.00 (0.000-2.50)
**Grand Total**	**1,008**	**1,622**	**19 (1.88)**	**45**	**1.18 (0.73-1.79)**

^#^ The maximum likelihood estimate (MLE) of flavivirus prevalence with a 95% confidence interval.

*Mosquitoes were pooled in the field without morphological identification.

### Genetic diversity and phylogenetic relationship of flaviviruses among mosquitoes

The NGS analysis identified contigs assigned to flaviviruses in 18 of the 19 libraries, with the exception of one “mini pool” (Mos_EL_69 in Table 2). Among these, complete ORF sequences were obtained from 13 libraries, corresponding to two “mini pools” of *Aedes*, four *Anopheles*, four *Culex*, and three *Uranoteania* mosquito species. The genome sizes of these sequences ranged from 10,649–11,018 nucleotides, with estimated mean read depths ranging from 60x to 1916x (Table 2). Phylogenetic analysis was performed using the amino acid sequences of the only complete polyproteins derived from these 13 libraries, alongside representative genomes from various genera within the *Flaviviridae* family, including *Orthoflavivirus*, *Pestivirus*, *Pegivirus*, and *Hepacivirus* (a total of 148 sequences from NCBI). All 13 sequences clustered within the genus *Orthoflavivirus*. The detected flaviviruses were further divided into two distinct groups: the sequence from Mos_L010, a pool of 10 *Culex vishnui* collected from caves, was closely related to TMUV, while the remaining 12 sequences, pools of one to five *Aedes*, *Anopheles*, *Culex*, and *Uranoteania* mosquito species also collected from caves, grouped with insect-specific flaviviruses (ISFVs) (S4 Fig).

**Table 2:** Characteristics of Flaviviruses-positive samples identified by NGS and sequencing analysis.

No.	Sample characteristics							NGS sequencing analysis metrics				
	NGS library ID	Species	Pool size	Date of collection	Habitat	Province	No. of raw paired reads	No. of contigs	No. of virus contigs	No. of flavivirus-assigned contigs	NT sequence length of samples with full ORF	Mean coverage per base of samples with full ORF
1	Mos_L010	*Culex vishnui*	10	August, 2023	Karstic cave	Khammouane	61,230,843	16,056	44	1	11,018	196
2	Mos_EL_69	*Culex hutchinsoni*	1	June, 2023	Village	Bolikhamxay	67,324,763	6,701	73	0	NA*	NA
3	Mos_EL_72	*Anopheles* sp.	1	July, 2023	Karstic cave	Bolikhamxay	82,325,371	48,068	77	3	10,884	415
4	Mos_EL_73	*Anopheles* sp.	3	June, 2023	Village	Bolikhamxay	79,031,021	13,543	78	5	10,823	642
5	Mos_EL_75	*Anopheles* sp.	5	June, 2023	Karstic cave	Bolikhamxay	111,773,336	24,177	49	3	10,870	233
6	Mos_EL_76	*Armigires kesseli*	4	June, 2023	Village	Bolikhamxay	85,296,342	36,910	191	69	NA	NA
7	Mos_EL_78	*Anopheles* sp.	5	June, 2023	Karstic cave	Bolikhamxay	81,964,035	34,430	65	3	10,699	96
8	Mos_EL_105	*Uranotaenia metatarsata*	4	August, 2023	Karstic cave	Khammouane	84,895,953	1,524	20	1	10,777	470
9	Mos_EL_106	*Aedes* sp. ([*Co*.] species1)	1	August, 2023	Forest	Khammouane	84,463,414	35,132	198	125	10,709	960
10	Mos_EL_107	*Culex vishnui*	1	July, 2023	Karstic cave	Vientiane	99,109,934	64,269	72	1	10,905	1,609
11	Mos_EL_108	*Culex vishnui*	1	July, 2023	Karstic cave	Vientiane	74,491,769	3,485	32	4	NA	NA
12	Mos_EL_109	*Uranotaenia metatarsata*	1	August, 2023	Karstic cave	Vientiane	72,852,408	44,163	270	109	10,684	60
13	Mos_EL_110	*Uranotaenia metatarsata*	1	August, 2023	Karstic cave	Vientiane	70,723,572	43,436	186	86	10,827	143
14	Mos_EL_111	*Uranotaenia longirostris*	1	August, 2023	Karstic cave	Vientiane	75,200,715	38,973	244	88	NA	NA
15	Mos_EL_112	*Culex quinquefasciatus*	1	August, 2023	Village	Vientiane	74,692,234	29,906	129	2	11,003	1,916
16	Mos_EL_113	*Aedes* sp.	1	August, 2023	Village	Vientiane	86,285,421	58,787	275	122	NA	NA
17	Mos_EL_114	*Aedes* sp. ([*Co*.] species1)	1	December, 2023	Karstic cave	Vientiane	75,561,670	4,936	33	8	10,649	1,136
18	Mos_EL_115	*Culex whitmorei*	2	August, 2023	Karstic cave	Khammouane	126,481,027	5,336	60	5	NA	NA
19	Mos_EL_116	*Culex vishnui*	1	July, 2023	Karstic cave	Vientiane	64,789,739	9,621	24	2	10,881	209

NOTE: NA*: not applicable.

**Fig 2:**
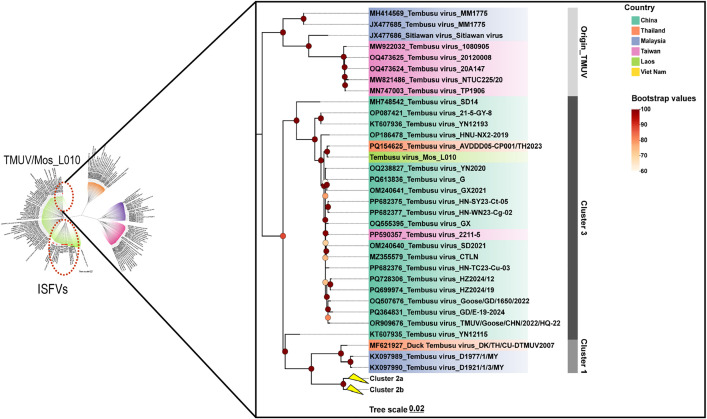
A maximum-likelihood phylogenetic tree of complete Tembusu virus (TMUV) genome. The evolutionary relationship of the TMUV/Mos_L010 highlighted in green. The tree includes representative strains from various clusters and regions for comparison. It was built using IQ-TREE with the GTR + F + I + R3 substitution model and 1,000 ultrafast bootstrap replicates. The scale bar represents the number of nucleotide substitutions per site.

To investigate the evolutionary relationship of the TMUV identified from Mos_L010 (referred to as TMUV/Mos_L010), a phylogenetic analysis was conducted using 216 representative sequences available in GenBank, covering regions including China, Thailand, Vietnam, Taiwan and Malaysia. The analysis indicated that TMUV/Mos_L010 clustered within Cluster 3, with bootstrap values exceeding 70%. Within this cluster, TMUV/Mos_L010 grouped with strains from Thailand (PQ154625) and China (OQ238827, PQ613836, and OM240641). Notably, TMUV/Mos_L010 formed a subclade with the Thai strain PQ154625, with>99% bootstrap support (Fig 2). To expand our analysis beyond complete genome sequences, we performed phylogenetic inference using the envelope (E) gene, which allowed inclusion of additional Thai strains. The E gene phylogeny confirmed the same clustering pattern, with TMUV/Mos_L010 grouping with Thai strain (AVDD05-CP001/TH202311) (S5 Fig). Notably, the expanded Thai dataset showed a broader evolutionary relationship between Thai and Laotian strains, with Thai strain P73_TH_2019 (OQ543571) branching basally to the TMUV/Mos_L010 and AVDD05-CP001/TH202311 subclade (S5 Fig).

**Fig 3:**
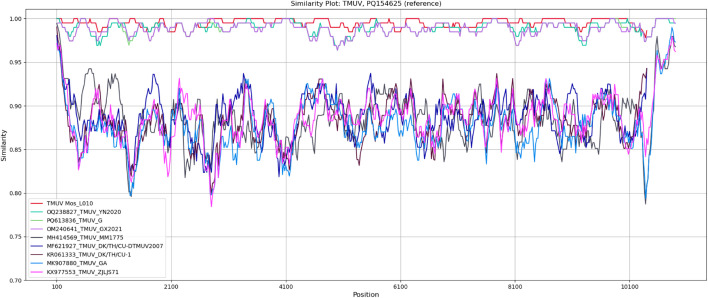
Similarity plot analysis of TMUV/Mos_L010 with reference TMUV genomes. The plot was generated using complete genome sequence of TMUV strain AVDD05-CP001/TH2023 (PQ154625) as a reference. TMUV/Mos_L010 and reference TMUV genome sequences were compared using the Kimura 2-parameter model with a sliding window of 200 nucleotides and a step size of 20 nucleotides.

These findings were corroborated by similarity plot analysis, which demonstrated that TMUV/Mos_L010 and the reference sequence (PQ154625) shared the highest sequence homology compared to other sequences (OQ238287, PQ261836, OM240641, MH414969, MF621297, KR061333, MK907880, and KX977553) (Fig 3). Consistently, sequence similarity analysis of the complete ORF indicated that TMUV/Mos_L010 shared 99.0% nucleotide and 99.7% amino acid homology with PQ154625 (S6 Fig).

A comparative alignment of the TMUV/Mos_L010 amino acid sequences was performed with ten reference sequences (JX477686, MH414569, MN747003, MF621927, KX686572, JF270480, MK542820, OQ238827, PQ613836, and PQ154625) to study amino acid polymorphisms. The TMUV/Mos_L010 strain exhibited unique single amino acid substitutions at the positions F491L, K860R, and D2288G. Additionally, the TMUV/Mos_L010 strain possessed two identical amino acid substitutions, at the positions M1168V and L2080I, compared to the Thai AVDD05-CP001/TH202311 (PQ154625) strain. The TMUV/Mos_L010 strain didn’t share previously reported substitutions at positions 154, 156, and 326 of the E protein, associated with the infectivity of the virus and transmission between ducks [43–45] (S3 Table).

**Fig 4:**
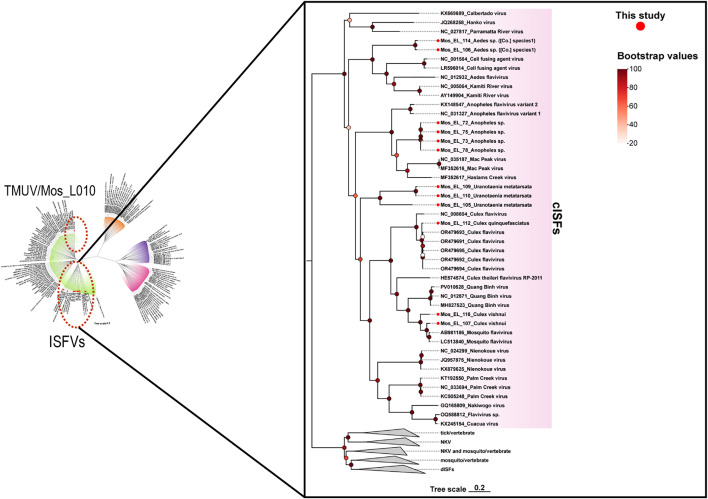
Phylogenetic analysis of detected insect-specific flaviviruses (ISFVs). A maximum-likelihood phylogenetic tree was made using the IQ-Tree based on nucleotide sequence alignment of ISFV complete genomes, identified in this study, and GenBank’s reference genomes from different vectors. The analysis was performed using the SYM + I + R7 substitution model and 1,000 ultrafast bootstrap replicates. Viruses identified in this study are marked with red dots preceding their taxon names, and the associated mosquito species are shown in italics following each taxon name. The scale bar indicates the number of nucleotide substitutions per site.

To gain a deeper understanding of the evolution of insect-specific flaviviruses (ISFVs) identified in this study, a phylogenetic reconstruction was conducted. This analysis included representative flaviviruses from various groups, as follows: classical insect-specific flaviviruses (cISFVs), no-known-vector flaviviruses (NKV), dual-host associated insect-specific flaviviruses (dISFVs), mosquito/vertebrate, and tick/vertebrate flaviviruses. The ISFVs detected clustered within the cISFV lineage with high sequence identity, supported by bootstrap values exceeding 99% (Fig 4).

Within this lineage, the ISFVs were subdivided into five distinct groups: (1) Two ISFVs (sample ID: Mos_EL_114 and Mos_EL_106) from *Aedes* mosquito species ([*Co.*] species1) clustered with the Cell Fusing Agent, *Aedes flavi*, and Kamiti River viruses, supported by >99% bootstrap confidence. (2) Four ISFVs (sample ID: Mos_EL_72, Mos_EL_73, Mos_EL_75, and Mos_EL_78) from *Anopheles* mosquito species grouped with Haslams Creek and Mac Peak viruses, with 70% bootstrap support. (3) Three ISFVs from *Uranotaenia metatarsata* clustered together in a separate group, where an ISFV (sample ID: Mos_EL_105) identified in Khammouane province was distinct from the other two ISFV (sample ID: Mos_EL_109 and Mos_EL_110) identified in Vientiane province. (4) One ISFV (sample ID: Mos_EL_112) from *Culex quinquefasciatus* clustered with other *Culex* flaviviruses, supported by more than 99% bootstrap confidence. (5) Two ISFVs (sample ID: Mos_EL_107 and Mos_EL_116) from *Culex vishnui* group grouped with Quang Binh and other mosquito flaviviruses, also supported by more than 99% bootstrap confidence.

### Genome organization of TMUV/Mos_L010 strain

**Fig 5:**
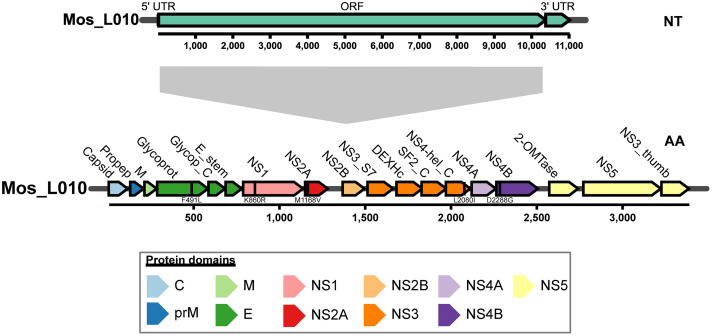
Genome organization of TMUV/Mos_L010. The ORF of LTMUV strain Mos_L010 was identified from NT sequence using NCBI ORF finder. Predicted protein domains were annotated based on the translated AA sequence using InterProScan. Gene annotations and corresponding coordinates indicated in S4 Table.

The TMUV/Mos_L010 genome consists of 11,018 nucleotides (nt), including an untranslated region (UTR) of 13 nt at the 5′ end, an ORF, and a 643 nt UTR at the 3′ end. The ORF encodes a single polyprotein, which is proteolytically processed into three structural proteins and seven nonstructural proteins. The structural proteins include the capsid (C; positions: 8–118 aa), and pre-membrane protein (prM), which is further subdivided into a pre-peptide (pr; positions: 133–211 aa) and the membrane protein (M; positions: 216–287 aa). The envelope (E) protein is comprised of three distinct domains: the central/dimerization domain (positions: 290–586 aa), the immunoglobulin-like domain (positions: 589–686 aa), and the stem/anchor domain (positions: 689–783 aa). The seven nonstructural proteins are NS1 (positions: 791–1140 aa), NS2A (positions: 1152–1284 aa), NS2B (positions: 1371–1498 aa), NS3, NS4A (positions: 2123–2264 aa), NS4B (positions: 2267–2509 aa), and NS5 (positions: 2575–3389). Notably, the NS3 protein (positions: 1516–2115 aa) is further organized into a protease domain (positions: 1516–1665 aa), a DEAD (Asp-Glu-Ala-Asp) domain (positions: 1683–1829 aa), and a C-terminal helical domain (positions: 1973–2115 aa). Similarly, the NS5 protein (positions: 2575–3389 aa) consists of a methyltransferase domain (positions: 2575–2743 aa), a fingers and palm domain (positions: 2774–3225 aa), and a thumb domain (positions: 3229–3389 aa) (Fig 5, S4 Table).

### Virus isolation

Although TMUV RNA was successfully detected and the complete coding sequence (CDS) obtained from one mosquito pool (Mos_L010), subsequent attempts to isolate the virus in BHK-21 and Vero E6 cell lines were unsuccessful. No cytopathic effect was observed after multiple blind passages, and viral RNA was not detectable in the culture supernatant.

## Discussion

Lao PDR, located in a heart of the Greater Mekong Subregion, is known for its rich biodiversity and considered a hotspot for infectious diseases [19]. The effects of globalization, cross-border trade with neighboring countries and land use expansion likely increase risks of infectious disease outbreaks in Lao PDR. Unfortunately, sustainable surveillance efforts are limited, leading to significant knowledge gaps regarding vector-borne pathogen circulation.

In this study, we conducted arbovirus surveillance by analyzing field-caught mosquitoes from six provinces in Lao PDR between 2021 and 2024. We identified over 100 mosquito species from 11 genera. From 19 detected flaviviruses, we obtained 13 coding-complete sequences. Phylogenetic analysis identified one TMUV strain from *Culex vishnui* mosquitoes, and 12 ISFV strains clustering into five phylogenetic groups from *Aedes*, *Anopheles*, *Culex*, and *Uranotaenia* mosquitoes. Other known pathogenic flaviviruses such as DENV, JEV, WNV, and ZIKV were not detected.

Notably, we detected TMUV in a pool of unfed *Culex vishnui* group mosquitoes collected from a karstic cave habitat near rice paddies in Khammouane Province during the rainy season (August). TMUV was first isolated from *Culex tritaeniorhynchus* in Malaysia in 1955 and has since been identified in other *Culex* species, including *Culex annulus, Cx. gelidus, Cx. pipiens,* and *Cx. vishnui*, across East and Southeast Asia, including Thailand, China, and Taiwan [10,44,46–49]. These findings suggest that Culex mosquitoes may serve as primary vectors and reservoir hosts for TMUV. Consistently, a study in Thailand’s Nan Province also reported the detection of TMUV in Culex mosquitoes along ecological gradients, including forests, lowland paddy fields, and peri-urban areas, emphasizing their role in maintaining the virus circulation in rural and peri-urban areas [50]. Furthermore, the close association of this mosquito group with water-intensive farming practices [51] highlights the persistence of TMUV in rice-growing regions and could represent a risk factor for exposure. Interestingly, our detection of TMUV in *Culex vishnui* group mosquitoes from karstic cave suggests a broader ecological range for host-seeking behaviours within this mosquito group, which may underscore their importance in pathogen exchange between different environments. Despite this ecological context, it should be noted that the morphological complexity of the *Culex vishnui* group poses challenges for accurate species identification, which has direct implications for TMUV surveillance and risk assessment. Diagnostic features to distinguish between *Culex vishnui* and *Cx. tritaeniorhynchus*, such as proboscis banding, are often variable or degraded in field specimens, leading to frequent misidentification [52,53]. These inaccuracies can be propagated into molecular databases, complicating phylogenetic studies and the understanding of TMUV transmission dynamics and insecticide resistance. Due to the pooled nature of our sample (n = 10), species-level genetic identification was not conducted in this study.

TMUV itself displays notable genetic diversity, comprising four major phylogenetic clusters. Our phylogenetic analysis placed the TMUV/Mos_L010 strain within cluster 3, which includes strains previously identified in China and Thailand. This expands the known geographic distribution of this cluster within Southeast Asia. TMUV/Mos_L010 shares a phylogenetic clade and >99% (bootstrap support) nucleotide identity with the TMUV strain AVDD05-CP001/TH2023 (PQ154625), which was recently associated with the death of bottlenose dolphins in Thailand [14]. The E gene phylogeny further revealed broader evolutionary connections between Thai and Laotian strains, with Thai strain P73_TH_2019 from Nan Province (OQ543571) [50] branching basally to the TMUV/Mos_L010 and AVDD05-CP001/TH202311 subclade (S5 Fig). This pattern suggests ongoing viral circulation between Thailand and Lao PDR. This finding gains additional significance from the recent surveillance data from China. Yang et al. (2026) documented an ecological shift in Guangdong Province, where cluster 2, the historically dominant lineage in China and Southeast Asia, was being replaced by cluster 3. Their late-2024 surveillance showed 32% positivity among tested samples, with all characterized strains belonging exclusively to cluster 3 [54]. Our detection of cluster 3 in Lao PDR, a country sharing borders with both China and Thailand, supports the hypothesis that this lineage is undergoing broad geographical expansion across mainland Southeast Asia. Notably, cluster 3 appears genetically closer to mosquito-origin lineages than the duck-adapted cluster 2. This evolutionary characteristic may partially explain our detection of cluster 3 in *Culex* mosquitoes, suggesting that this lineage may maintain more efficient avian-mosquito transmission cycles. Such enhanced vector competence could facilitate both local persistence and regional spread through migratory bird movements. Future surveillance efforts should prioritize cross-border sites in this region to identify the source of transboundary TMUV transmission, while also implementing cluster-specific monitoring to track the progression of this ecological turnover.

The high percentage of sequence homology at the nucleotide level raises the possibility that TMUV/Mos_L010 may also have pathogenic potential for wild animal hosts. Notably, two amino acid substitutions (M1168V and L2080I) were shared between the two strains and three additional substitutions (F491L, K860R and D2288G) were unique to TMUV/Mos_L010 (Fig 5 and S3 Table). However, the impact of these amino acid substitutions on infectivity among mammalian hosts remains unknown. Like AVDD05-CP001/TH2023, no mutations were observed in the E protein that have been previously associated with mammalian infections [14].

The detection of two nearly identical TMUV strains in distinct ecological niches and geographic locations from different animals (mosquito in this study), suggests potential long-distance virus dissemination, possibly mediated by the seasonal migration of infected birds. Infected mosquitoes may feed on both avian and mammalian hosts, facilitating cross-species transmission. Furthermore, TMUV may spread through alternative routes, such as direct contact or exposure to contaminated environments [55]. Contact with contaminated water could represent an additional transmission route, particularly for aquatic or semi-aquatic animals. Further research is needed to clarify transmission pathways.

Although TMUV outbreaks among poultry have been reported in China and other neighboring countries, to our knowledge, no outbreaks to date have been documented in Lao PDR. However, the detection of TMUV in Laotian mosquitoes, together with the presence of similar ecological conditions and competent vector species, suggests a potential risk of undetected transmission. This highlights the need for targeted surveillance in Lao PDR, especially in agricultural areas, particularly those associated with rice cultivation and poultry production. Furthermore, detection of TMUV RNA and antibodies in humans in China and Thailand [16,17] highlights the need for surveillance not only in mosquitoes and poultry but also among high-risk human populations, including farmers, poultry workers, and duck breeders, to understand host-pathogen transmission routes, transmission dynamics, and inform potential public health mitigation strategies.

Alongside TMUV, the detection of several ISFVs in this study expands our understanding of flavivirus diversity in Lao PDR and supports the notion that ISFVs are widely distributed geographically [56]. Five distinct ISFV groups were detected and phylogenetically clustered within the cISFVs lineage. While ISFVs are currently not known to infect vertebrates, they may play important roles in shaping the arbovirus ecology, potentially influencing mosquito immunity, vector competence, or even modulating co-infections with pathogenic flaviviruses [57,58]. Further studies are therefore needed to clarify the ecological and biological roles of ISFVs among vectors, hosts, and the environment.

This study has several limitations. First, attempts to isolate TMUV/Mos_L010 in cell culture were unsuccessful. Virus isolation was attempted between June and July, approximately 22 months after the mosquitoes were collected in August 2023 and stored at −80°C. The lack of success was likely due to a combination of the prolonged storage period and delays between mosquito death in the CDC light traps and field processing (often 12–17 hours), both of which may have resulted in loss of virus viability. Consequently, no investigations could be conducted to evaluate a potential role of the detected mutations in enhancing or limiting the infectivity of TMUV/Mos_L010 in mammalian cells or hosts. In addition, isolation attempts were limited to BHK-21 and Vero E6 cell lines, as our primary focus was vertebrate infection. However, the use of insect cell lines (e.g., C6/36) might have increased detection sensitivity for TMUV propagation. An alternative approach that may increase the chance of virus isolation is the collection of mosquito excreta [59], which may provide higher viral concentrations with reduced microbial contamination. Incorporating such field-deployable, excreta-based methods, together with timely sample processing, may improve virus isolation success in future TMUV surveillance and facilitate better assessment of the pathogenic potential of TMUV/Mos_L010. Second, mosquitoes were collected and analyzed as pooled samples, which limited species-level resolution and prevented the identification of species-specific infection patterns. In addition, sample collection was not uniformly conducted across all locations. Some sites were sampled only once, whereas others were sampled multiple times. This uneven sampling effort may have introduced bias and limited direct comparability between sites. Third, trap selection may have influenced the mosquito species obtained and subsequent virus detection. This study primarily used CDC light traps, which are effective for collecting many phototactic mosquito species. However, the use of other trap types, such as BG-Sentinel or BG-Pro traps, which incorporate additional attractants (e.g., visual cues, CO₂, and host kairomones), could improve representation of other mosquito taxa and increase the likelihood of detecting viruses associated with different vectors in future studies. Finally, only 13 complete ORF sequences were recovered from the 19 positive samples, resulting in an incomplete characterization of the genetic diversity and evolutionary dynamics of the detected viruses.

## Conclusion

This study described previously undocumented flavivirus diversity in Lao PDR, identifying a new TMUV strain (TMUV/Mos_L010) that is phylogenetically similar to a strain pathogenic to dolphins in Thailand. This discovery expanded the geographical range of TMUV cluster 3 species to include Lao PDR within the Indo Pacific region. The presence of multiple ISFVs in mosquitoes highlighted the rich and previously underexplored mosquito virome in the area, pointing to a need for improved arbovirus surveillance. Understanding the evolution and transmission dynamics of flaviviruses is critical for protecting local populations, travelers and military personnel, ensuring operational readiness in affected regions. Prioritizing vector-pathogen surveillance is essential for maintaining biosecurity in the Indo-Pacific region.

## Supporting information

S1 Table Main characteristic of collection sites for mosquitoes in Lao PDR. (XLSX)

S2 Table A total number of mosquito species from different types of habitats. (XLSX)

S3 Table Comparative analysis of amino acid substitutions in the TMUV/Mos_L010 strain and other representative strains. (XLSX)

S4 Table Protein domain identification and corresponding coordinates of TMUV/Mos_L010 strain. (XLSX)

S1 Fig Overall species composition by month, district, and province. (TIF)

S2 Fig Species composition of Aedes, Anopheles, and Culex by month, district, and province. (TIF)

S3 Fig Species composition of other genera by month, district, and province. (TIF)

S4 Fig Phylogenetic analysis of detected flaviviruses with 148 sequences downloaded from NCBI. An unrooted maximum-likelihood phylogenetic tree was reconstructed with IQ-TREE based on aligned amino acid sequences of coding-complete genomes of flaviviruses detected in this study and representative flaviviruses from different genera. The tree was constructed using the LG + F + R6 substitution model and 1,000 ultrafast bootstrap replicates. Viruses identified in this study are marked with red dots preceding their taxon names. The scale bar indicates the number of substitutions per site. TMUV: Tembusu virus, ISFV: Insect specific virus.
(TIF)

S5 Fig Phylogenetic analysis based on the envelope (E) gene of TMUV. Inclusion of additional Thai strains confirmed the clustering pattern observed in whole-genome analysis, with TMUV/Mos_L010 grouping closely with the Thai strain AVDD05-CP001/TH202311.
(TIF)

S6 Fig Complete open reading frame (ORF) identity matrices of TMUV/Mos_L010 and other 29 representative TMUV sequences. (TIF)
