# Supplementary figures and images for: Detection and genetic characterization of Tembusu virus and other flaviviruses from mosquitoes in Lao PDR
Source: PLoS One. 2026 Jun 8;21(6):e0351023. doi: 10.1371/journal.pone.0351023 (PMC13245760; doi:10.1371/journal.pone.0351023)

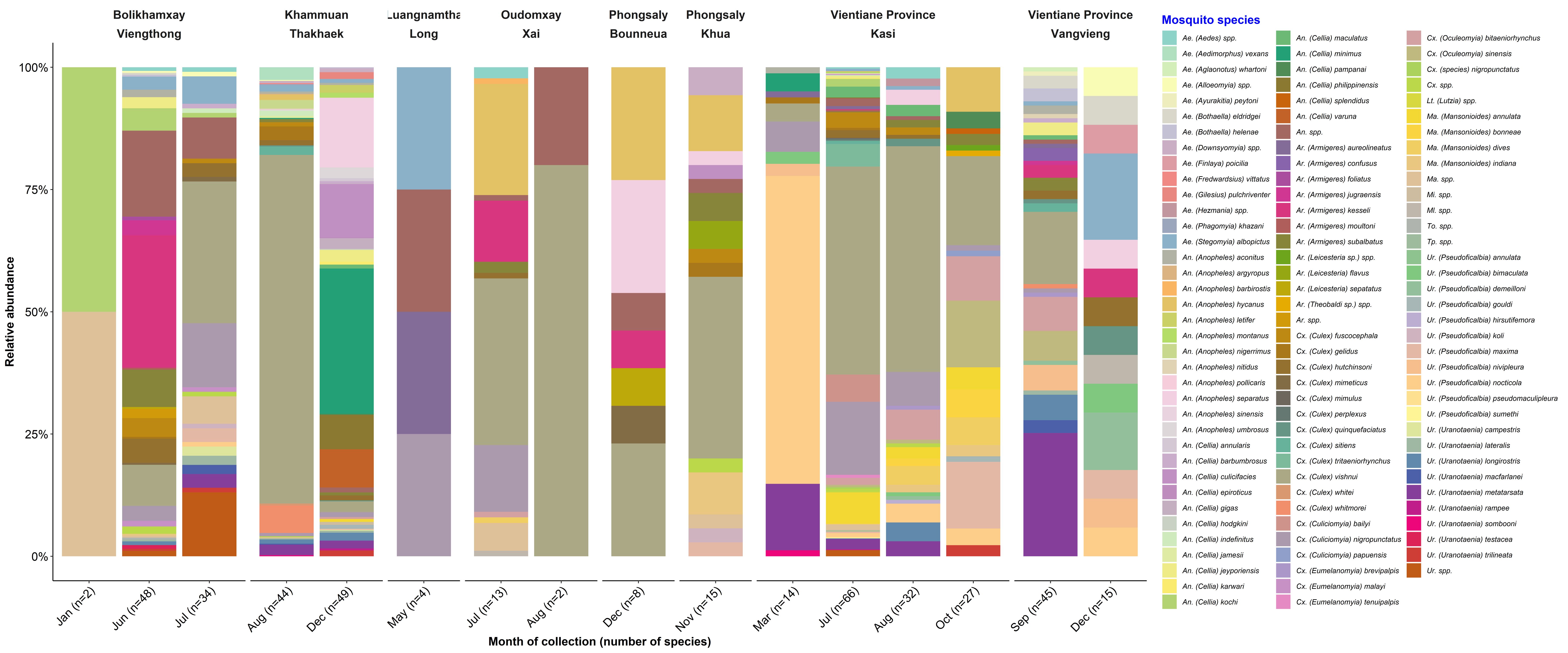

Supplement: S1 Fig — (TIF) [file pone.0351023.s005.tif]

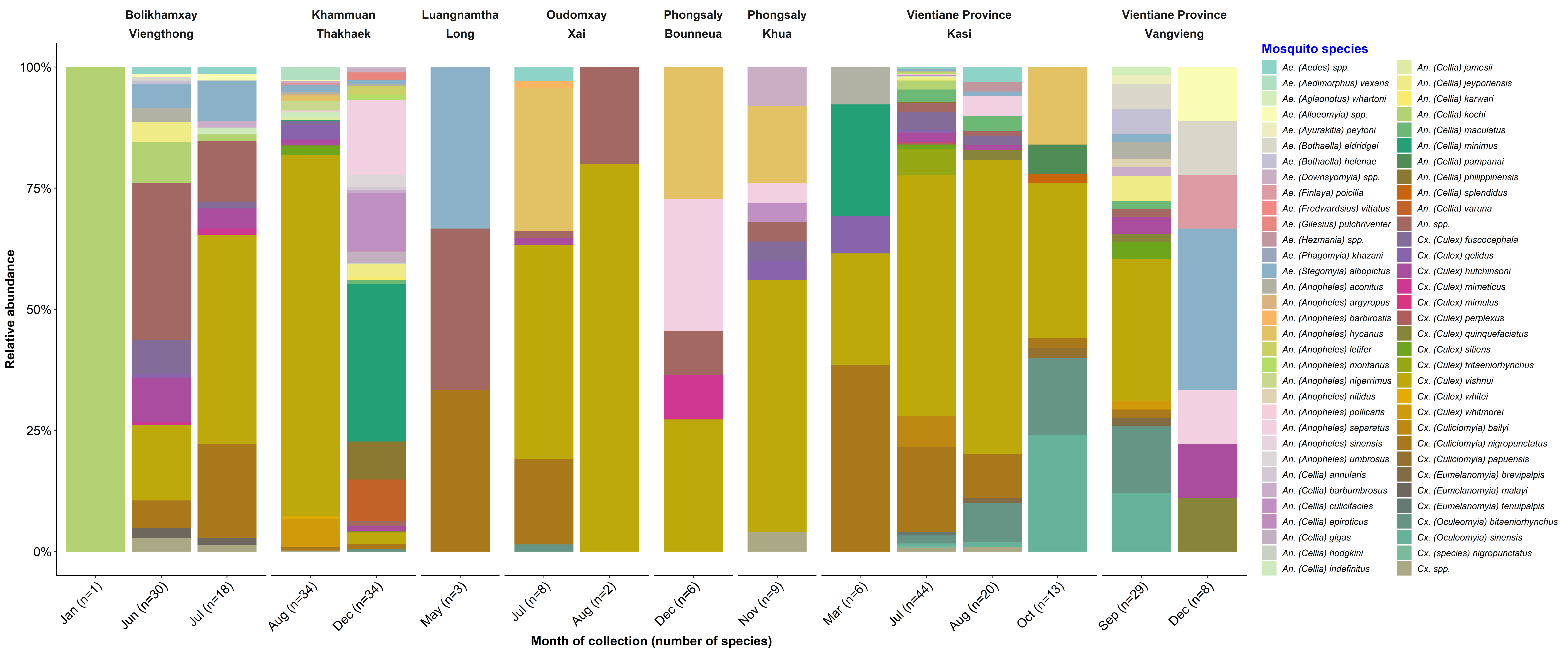

Supplement: S2 Fig — (TIF) [file pone.0351023.s006.tif]

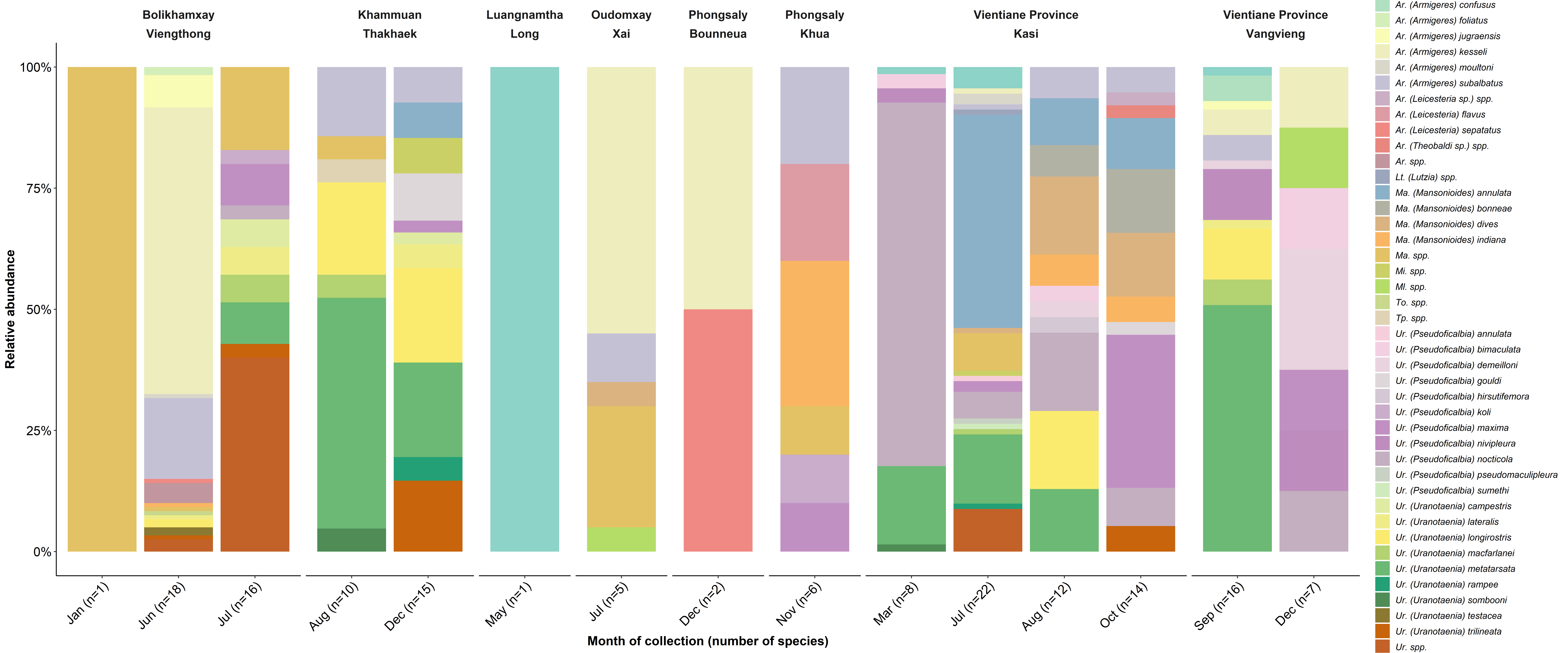

Supplement: S3 Fig — (TIF) [file pone.0351023.s007.tif]

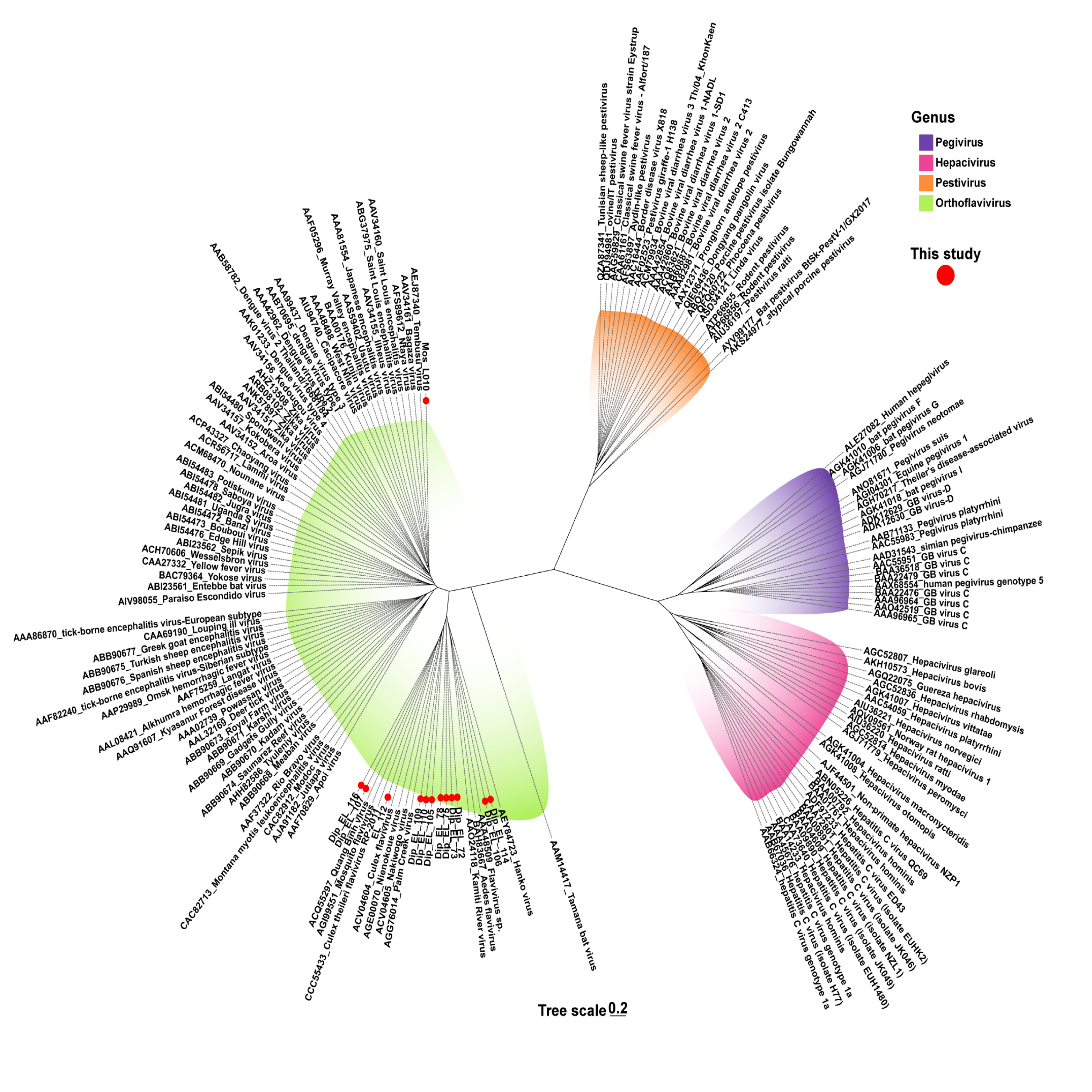

Supplement: S4 Fig — An unrooted maximum-likelihood phylogenetic tree was reconstructed with IQ-TREE based on aligned amino acid sequences of coding-complete genomes of flaviviruses detected in this study and representative flaviviruses from different genera. The tree was constructed using the LG + F + R6 substitution model and 1,000 ultrafast bootstrap replicates. Viruses identified in this study are marked with red dots preceding their taxon names. The scale bar indicates the number of substitutions per site. TMUV: Tembusu virus, ISFV: Insect specific virus. (TIF) [file pone.0351023.s008.tif]

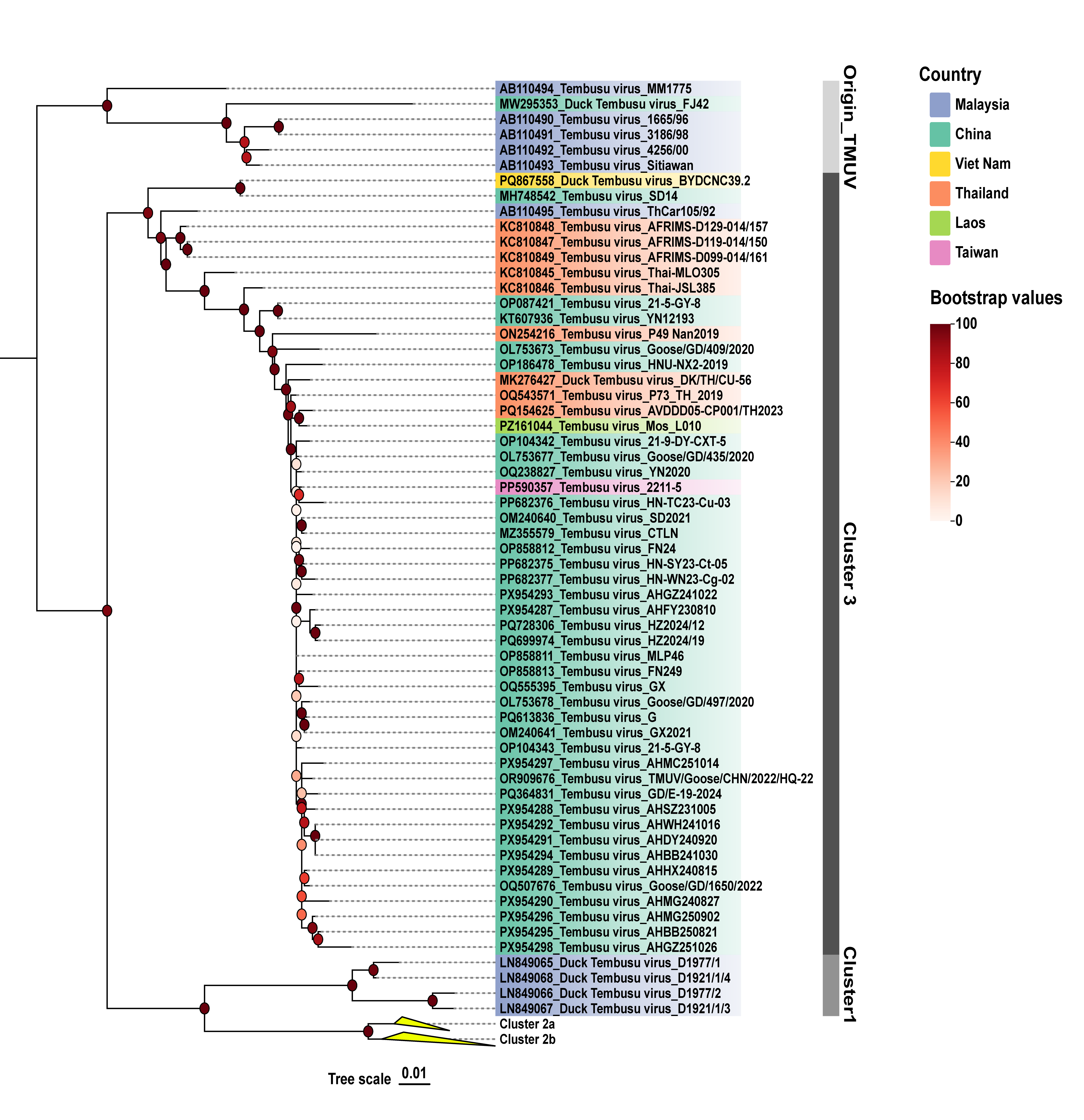

Supplement: S5 Fig — Inclusion of additional Thai strains confirmed the clustering pattern observed in whole-genome analysis, with TMUV/Mos_L010 grouping closely with the Thai strain AVDD05-CP001/TH202311. (TIF) [file pone.0351023.s009.tif]

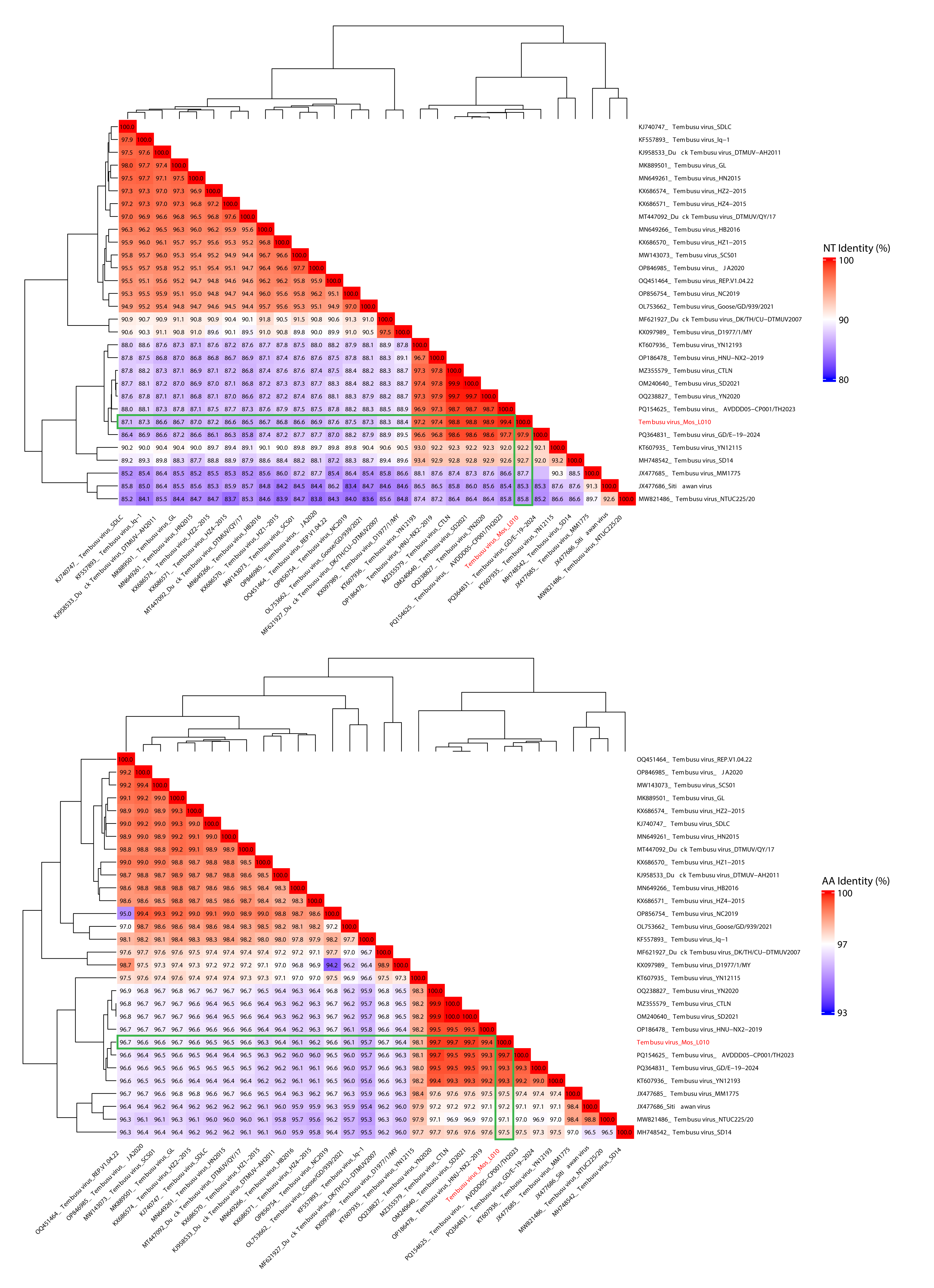

Supplement: S6 Fig — (TIF) [file pone.0351023.s010.tif]
